# Development of Microdroplet Generation Method for Organic Solvents Used in Chemical Synthesis

**DOI:** 10.3390/molecules25225360

**Published:** 2020-11-17

**Authors:** Shohei Hattori, Chenghe Tang, Daiki Tanaka, Dong Hyun Yoon, Yoshito Nozaki, Hiroyuki Fujita, Takashiro Akitsu, Tetsushi Sekiguchi, Shuichi Shoji

**Affiliations:** 1 Department of Electronic and Physical Systems, School of Fundamental Science and Engineering, Waseda University, 3-4-1 Okubo, Shinjuku-ku, Tokyo 145-0065, Japan; c_tang@shoji.comm.waseda.ac.jp (C.T.); shojis@waseda.jp (S.S.); 2 Research Organization for Nano & Life Innovation, Waseda University, 5-1-3 Waseda-tsurumakicho, Shinjuku-ku, Tokyo 162-0041, Japan; d.tanaka@ruri.waseda.jp (D.T.); yoon@shoji.comm.waseda.ac.jp (D.H.Y.); y.nozaki@aoni.waseda.jp (Y.N.); t-sekiguchi@waseda.jp (T.S.); 3 Advanced Research Laboratory, Canon Medical Systems Corporation, 1385 Shimoishigami, Otawara-shi, Tochigi 324-0036, Japan; hiroyuki12.fujita@medical.canon; 4 Department of Chemistry, Faculty of Science, Tokyo University of Science, 1-3 Kagurazaka, Shinjuku-ku, Tokyo 162-0825, Japan; akitsu2@rs.tus.ac.jp

**Keywords:** microfluidics, microdroplets, organic solvents, organic droplets

## Abstract

Recently, chemical operations with microfluidic devices, especially droplet-based operations, have attracted considerable attention because they can provide an isolated small-volume reaction field. However, analysis of these operations has been limited mostly to aqueous-phase reactions in water droplets due to device material restrictions. In this study, we have successfully demonstrated droplet formation of five common organic solvents frequently used in chemical synthesis by using a simple silicon/glass-based microfluidic device. When an immiscible liquid with surfactant was used as the continuous phase, the organic solvent formed droplets similar to water-in-oil droplets in the device. In contrast to conventional microfluidic devices composed of resins, which are susceptible to swelling in organic solvents, the developed microfluidic device did not undergo swelling owing to the high chemical resistance of the constituent materials. Therefore, the device has potential applications for various chemical reactions involving organic solvents. Furthermore, this droplet generation device enabled control of droplet size by adjusting the liquid flow rate. The droplet generation method proposed in this work will contribute to the study of organic reactions in microdroplets and will be useful for evaluating scaling effects in various chemical reactions.

## 1. Introduction

Microfluidic technology allows the manipulation of minute volumes of fluids in micro/nanoscale flow channels. On the micro/nanoscale, certain physical properties become more influential, causing fluids to exhibit behavior distinct from that of macroscopic fluids. In particular, the characteristics of micro/nanoscopic fluids are dominated by three phenomena, namely, highly efficient mass and heat transfer, a strong influence of viscosity, and pronounced surface effects [1,2,3]. In addition, microfluidic systems permit systematic control over and manipulation of fluids and fluid interfaces [4]. Consequently, microfluidic technology has recently attracted considerable attention, especially in the fields of chemistry [5,6], biology [7,8], medicine [9], and engineering [10].

Channel-based droplet microfluidics is a subfield of microfluidics involving the generation and manipulation of droplets in microchannels. Such droplets are typically generated using immiscible multiphase fluids composed of a continuous phase (carrier) and dispersed phase (droplets) and are systematically manipulated via passive or active methods [11,12]. To date, a variety of droplet manipulation techniques have been devised to permit sorting [13], merging [14], splitting [15], mixing [16], and trapping [17] of droplets. The combination of these methods has led to the development of microscale droplet reactors with numerous potential biomedical [18,19,20] and chemical [21] applications. These reactors enable laboratory operations such as mixing reagents, transporting samples, and performing observation or analysis of target samples on a micro scale.

For chemical reactions and analysis, microscale droplet reactors are effective and efficient experimental tools. They show enhanced mixing and mass transfer within short diffusion distances and therefore can shorten reaction times [22,23,24,25]. They also allow for fast heat transfer and can reduce response times for controlling reaction temperatures. Droplets serve as small-volume containers in which reactions can be isolated. They are monodispersed and can be generated extremely rapidly. Therefore, microscale droplet reactors are suitable for quantitative studies as well as high-throughput experiments. In addition, they offer the possibility of performing many reactions in parallel using a single chip [26]. However, microdroplet techniques have predominantly been applied to aqueous-phase reactions rather than reactions in organic solvents. High reactivity organic reactions require strict temperature control and low reactivity organic reactions requires long experimental time of several to dozens of hours [27]. Microdroplet methods have the potential to eliminate the need for temperature control due to fast heat diffusion and accelerate reactions due to fast mass transfer. Although the advantages of organic-phase reactions using microdroplets have been demonstrated [28], examples of such reactions remain very limited.

The reason why microdroplet methods have not yet been widely used in studies of organic-phase reactions is that the physical and chemical properties of organic solvents are not compatible with conventional microfluidic devices. There are two key challenges in the generation of organic solvent droplets. The first is damage to device materials by organic solvents. The second is the difficulty of selecting a combination of carrier and organic solvents that can form stable monodisperse droplets. With respect to the chemical resistance of device materials, microfluidic devices are typically fabricated using soft lithography techniques and are composed of polydimethylsiloxane (PDMS) [29]. However, PDMS swells upon contact with organic solvents, potentially damaging the channel pattern [30]. To avoid swelling, resin devices with high chemical resistance, including a thiol–ene-based resin device, have been developed [31]. However, swelling in these devices was caused by some organic solvents, such as acetone and dichloromethane, despite the application of long-term heat treatment to increase their chemical resistance. In addition, thiol–ene-based resin shrinks during the curing process and expands during heating [32,33], which lowers the accuracy of the channel pattern. Consequently, devices fabricated from physically stable materials with high chemical resistance such as silicon and glass are essential. With respect to the combination of carrier and organic solvents, two important properties are low solubility of the organic solvent in the carrier and the relative wettability of the carrier and organic solvents on the channel surface. Even if the carrier and the organic solvent are immiscible, if the organic solvent has higher wettability than the carrier on the channel surface, then droplets will not form. Therefore, for each organic solvent, the carrier should be carefully selected with consideration given to its miscibility with the organic solvent and its wettability on the channel surface. For these reasons, it has not yet been possible to apply microdroplet techniques to organic-phase reactions despite their expected utility in the field of organic chemistry. Therefore, the development of methods for generating droplets of organic solvents is urgently needed.

In this work, droplet generation was successfully achieved for five typical organic solvents frequently used in organic synthesis, namely, toluene, chloroform, methanol, tetrahydrofuran (THF), and dimethyl sulfoxide (DMSO). Droplet generation for some organic solvents incompatible with resins, namely, acetone, acetonitrile, and dichloromethane, was also confirmed. Combinations of organic solvents and continuous-phase solutions that do not mix with each other and are suitable for droplet generation were determined. These combinations were selected with consideration of miscibility and ease of modifying wettability on the channel surface and were then used to generate stable droplets of the organic solvents in a silicon/glass-based device. The inertness of the device materials precluded the possibility of channel swelling. Droplet size and droplet generation rate are important parameters for the application of microdroplet methods to chemical reactions and should thus be controllable. Accordingly, the variation of droplet size and droplet generation rate under different flow rates of the dispersed and continuous phases were also evaluated.

## 2. Results

### 2.1. Droplet Generation of Typical Organic Solvents

**Figure 1:**
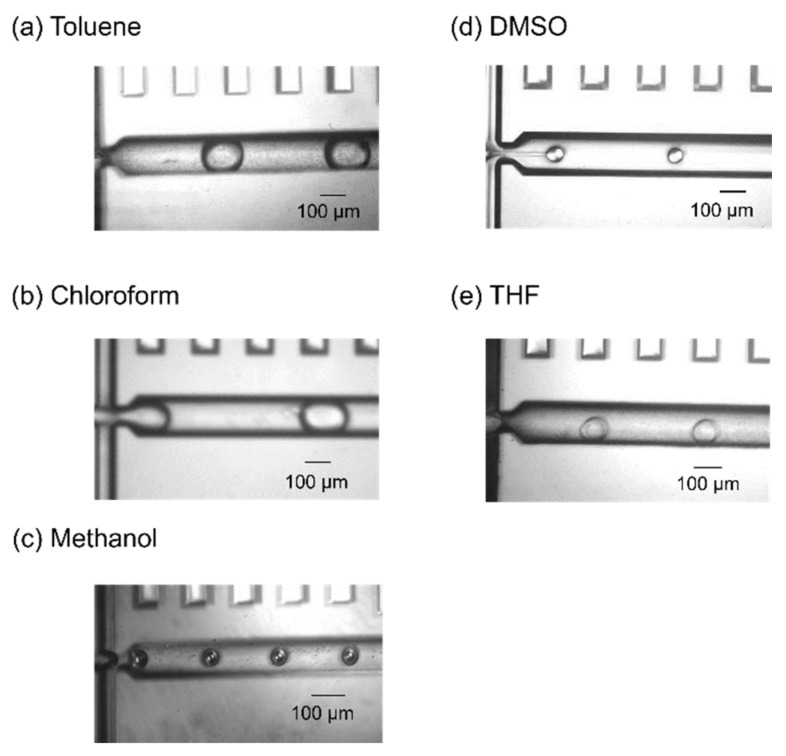
Images showing the microfluidic flow at the cross-junction area for (**a**) toluene, (**b**) chloroform, (**c**) methanol, (**d**) dimethyl sulfoxide (DMSO), and (**e**) tetrahydrofuran (THF). The flow rates of the dispersed and continuous phases (µL/min) were (**a**) 1 and 3, (**b**) 1 and 3, (**c**) 0.3 and 3, (**d**) 0.4 and 8, and (**e**) 0.5 and 5, respectively. The magnification of microscope was 10×.

**Figure 2:**
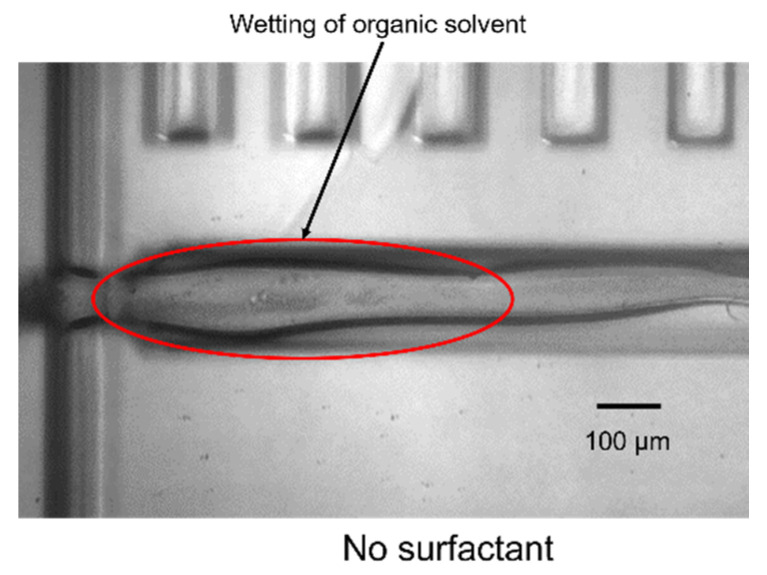
Image showing the laminar flow of toluene in the absence of surfactant. The flow rates of the dispersed and continuous phases (µL/min) were 1 and 3, respectively. The magnification of microscope was 10×.

Figure 1 presents images of the cross-junction area of the silicon/glass-based devices and shows the flows of the five combinations of dispersed-phase and continuous-phase solutions. For all five organic solvents, the dispersed-phase solution flowed through the channel under laminar flow when the carrier solution did not have enough encapsulation ability, as shown in Figure 2 for toluene as a representative example. In contrast, when the carrier solution had sufficient encapsulation ability, monodisperse droplets of the organic solvents were continually generated. The flow rate at which droplets were generated was dependent on the combination of the dispersed and continuous phases (Table 1). The standard deviation of droplet length was as low as 4.3% at most and the droplet generation rate ranged from 9.55 to 23.9 Hz. No deformation of the device channels was observed, and stable droplet generation of toluene and chloroform continued for several hours whereas that of methanol and DMSO continued for about an hour. THF droplet generation continued for tens of minutes. However, methanol, DMSO, and THF gradually wetted the channel surface. This was due to a change in wettability. Wetting by methanol and DMSO was likely because the hydrophobic treatment wore off over time. Wetting by THF was likely because THF and saturated saline separate into two phases but are not completely immiscible (Table 3), and THF gradually contacted the channel surface. Therefore, an improved hydrophobic treatment method and a dispersed phase solution with lower solubility in THF are needed for long-term methanol, DMSO, and THF droplet generation. The generated droplets of toluene and chloroform were passed through the channel to the outlet without collapsing. Toluene and chloroform droplets could be transported to the pool consisting of carrier solution and oil and did not collapse for at least 30 min. These results indicate that a surfactant was helpful for generating and stabilizing organic solvent droplets, as in the case of water-in-oil and oil-in-water droplets [34]. However, methanol, DMSO, and THF droplets sometimes merged at the outlet or collapsed after transportation. This was due to insufficient optimization of the surfactant concentration. Accordingly, stable droplet generation was successfully realized for five common organic solvents using immiscible continuous-phase solutions and surfactants with appropriate hydrophile-lipophile balance (HLB) values. 

### 2.2. Droplet Generation of Organic Solvents Incompatible with High Chemical Resistance Resins

**Figure 3:**
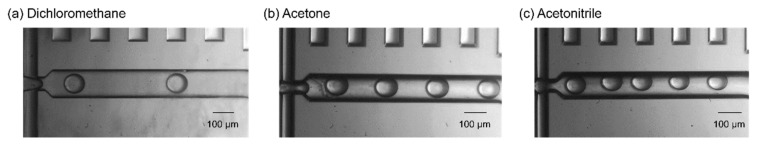
Images showing the microfluidic flow at the cross-junction area for (**a**) dichloromethane, (**b**) Acetone, (**c**) Acetonitrile. The flow rates of the dispersed and continuous phases (µL/min) were (**a**) 1 and 10, (**b**) 1 and 3, and (**c**) 1 and 3, respectively. The magnification of microscope was 10×.

To confirm the high chemical resistibility of silicon/glass-based devices, fluid experiments with acetone, acetonitrile, and dichloromethane, which swelled thiol–ene-based devices, were also conducted. Figure 3 shows the successful generation of acetone, acetonitrile, and dichloromethane droplets, which were generated at the cross-junction area of the channel, as is the case with typical organic solvents. The standard deviation of droplet length was as low as 3.1% at most and the droplet generation rate ranged from 27.3 to 47.6 Hz. The droplet generation of these solvents was stable and continued for several hours. Dichloromethane droplets were passed through the channel without collapsing or merging at the outlet. However, acetone and acetonitrile droplets sometimes merged in the channel due to the absence of surfactants. Table 2 shows the combinations of organic solvents and carrier solutions for acetone, acetonitrile, and dichloromethane droplet generation. For dichloromethane droplet generation, 1 wt % PVA solution was used as the carrier solution, which prevented the droplets from merging. However, surfactants could not be used for generating droplets of acetone and acetonitrile because they are miscible with many solvents. Accordingly, there were few carrier solution candidates. Although carrier liquids were found that were immiscible with acetone and acetonitrile and could generate droplets, surfactants that are soluble in the carrier liquids and have sufficient encapsulation ability have not yet been found. To prevent droplet merging, a proper surfactant is required, and thus further investigation is needed. Nevertheless, it was confirmed that the silicon/glass-based device has high chemical resistance and is capable of generating droplets of various organic solvents, according to these droplet generation results.

### 2.3. Droplet Size and Generation Rate Variation

**Figure 4:**
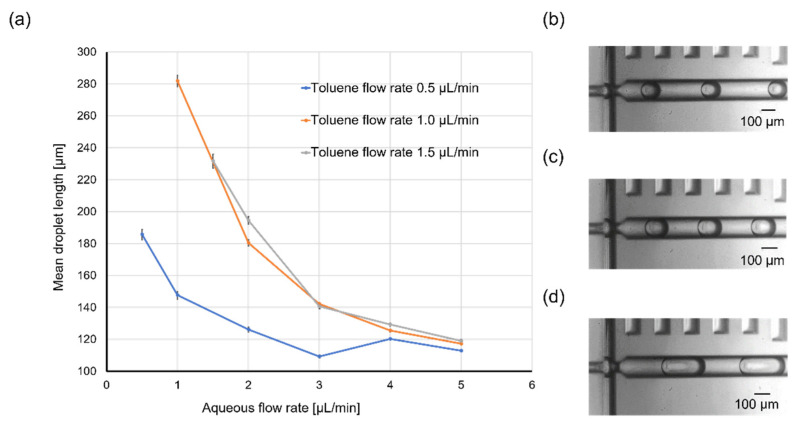
(**a**) Variation of the mean toluene droplet length under different flow rates of the dispersed phase (toluene) and continuous phase (water containing 4 wt % Tween 20). (**b**–**d**) Images of toluene droplets in the cross-junction area for flow rates of the organic and aqueous phases (µL/min) of (**b**) 1 and 5, (**c**) 1 and 3, and (**d**) 1 and 1, respectively. The sample size of the mean droplet measurement was 30 droplets and the error bars represent the standard error of the mean droplet length. The magnification of microscope was 10×.

Given that scaling effects are important in chemical reactions involving microdroplets, control over droplet size is desirable. Therefore, the influence of the flow rates of the two phases on droplet size was investigated using toluene as a representative example. Figure 4a shows the variation of the mean toluene droplet length under different flow rates of the dispersed phase (toluene; 0.5, 1.0, or 1.5 µL/min) and continuous phase (water containing 4 wt % Tween 20; 0.5, 1.0, 1.5, 2.0, 3.0, 4.0, or 5.0 µL/min). The mean droplet length ranged from 110 to just over 280 µm. As shown in the plot, the mean droplet length decreased with increasing flow rate of the aqueous phase. The flow rate of the organic phase had the opposite effect on the droplet length, with an increased flow rate affording larger droplets. Uniform droplet generation was also confirmed, especially for high total flow rates (i.e., the sum of the flow rates of the aqueous and organic phases); the standard deviation of the droplet length ranged from 10% at low total flow rates to 1% at high total flow rates. However, the variation of the mean droplet length was irregular when the toluene flow rate was 1.5 µL/min, which was ascribed to facile wetting of the device surface by toluene and inhibited droplet formation when the toluene flow rate was too high. Figure 4b–d presents images of toluene droplets in the cross-junction area for various aqueous flow rates and a fixed toluene flow rate. It can be clearly seen that the flow rate of the continuous phase affected the toluene droplet length. These results indicate that the toluene droplet length could be manually controlled by adjusting the flow rates of the organic and aqueous phases.

**Figure 5:**
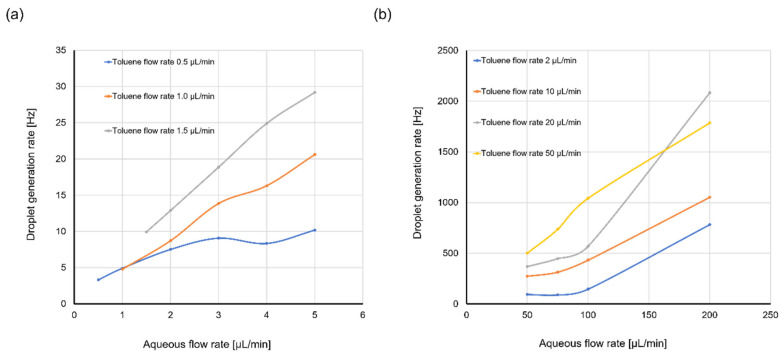
Variation in toluene droplet generation rate under different flow rates of dispersed phase (toluene) and continuous phase (water containing 4 wt % Tween 20). (**a**) The toluene flow rate was 0.5, 1.0, or 1.5 µL/min, and the aqueous flow rate was 0.5, 1.0, 1.5, 2.0, 3.0, 4.0, or 5.0 µL/min. (**b**) The toluene flow rate was 2, 10, 20, or 50 µL/min, and the aqueous flow rate was 50, 75, 100, or 200 µL/min.

The toluene droplet generation rate also changed as the flow rates were changed. Figure 5a,b shows the variation in toluene droplet generation rate under different flow rates of dispersed and continuous phases. It was shown that the droplet generation rate increased with the increasing toluene flow rate or aqueous flow rate. The generation rate ranged from about 3.3 to 2833.3 Hz. Sufficient throughput was achieved at high total flow rates, especially when the flow rate of continuous-phase was high. However, the generation rate of toluene droplets was lower than that of water-in-oil droplets. This was due to the low capillary number of the toluene/water system. The droplet generation rate increases as capillary number (*Ca*), which can be defined by the following Equation (1), increases [35].

(1) $$Ca=\frac{\mu_{c}u_{c}}{\sigma }$$

Here, $\mu_{c}$ and $u_{c}$ are the dynamic viscosity and the velocity of the continuous phase, respectively. *σ* is the surface tension between the dispersed and continuous phases. The viscosity of water with the surfactant was about 0.9 mPa s and the interfacial tension between toluene and water with the surfactant was 10 mN/m [36]. In contrast, the viscosity of mineral oil is 57.2 mPa s and the interfacial tension between water and oil with Span 80 is 5 mN/m. Therefore, the capillary number of a toluene-in-water droplet is significantly, approximately 100 times, lower than that of a water-in-oil droplet when the velocity of the continuous phase was the same. To improve throughput, a carrier solution having high viscosity and low interfacial tension with organic solvents will be needed. It was also found that droplet generation destabilized when the total flow rate was too high. This was because the capillary number increased with increasing aqueous flow rate and the generation regime changed from a dripping regime to a jetting regime [35]. Increasing the velocity of the carriers helps to increase throughput, but it must be increased with care to ensure that the generation regime does not change.

## 3. Materials and Methods

### 3.1. Chemicals and Sample Preparation

**Figure 6:**
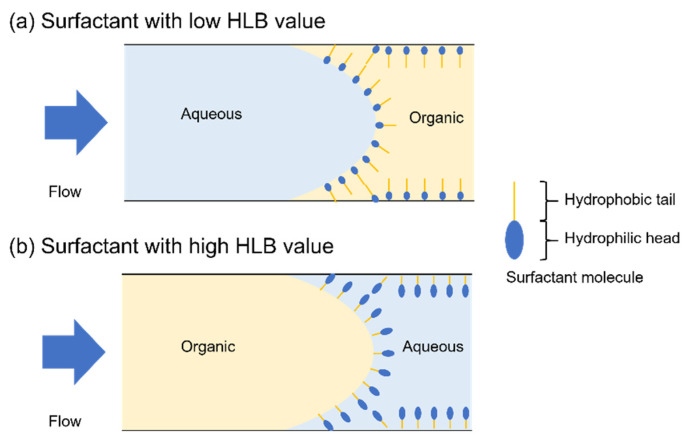
Interface of the aqueous and organic phases at the droplet generation area when using the surfactant with (**a**) a low HLB value and (**b**) a high HLB value.

Table 3 summarizes the combinations of continuous-phase and dispersed-phase solutions used in the microfluidic experiments. Organic solvents are typically classified into three types: nonpolar, protic polar, and aprotic polar. This classification scheme is important for chemical reactions and the appropriate organic solvent should be used for a particular target reaction. In this work, five common organic solvents belonging to these three classes and possessing different dipole moments were selected as the dispersed phase to evaluate the versatility of our droplet generation method; toluene and chloroform were used as nonpolar solvents, methanol was used as a protic polar solvent, and THF and DMSO were used as aprotic polar solvents. These solvents are used for some scientifically and industrially important reactions and a wide range of applications is expected. For example, diethyl ether and THF are used as solvents for the Grignard reaction, and toluene is used for the Wittig reaction. Methanol is used as a solvent for synthesis of metal complex proteins that are used as drugs. Furthermore, three organic solvents causing strong damage to resins (acetone, acetonitrile, and dichloromethane) were also selected as the dispersed phase to confirm the high chemical resistance of the silicon/glass-based device. All eight solvents were purchased from Suzuki Chemical Industry (Okazaki, Japan). Liquids that were immiscible with the dispersed-phase solutions were selected as the continuous phase. To select the carrier, simple preliminary experiments were conducted. One of the five organic solvents and a carrier candidate were poured into a glass vial. If the carrier candidate and organic solvent were immiscible, they would clearly separate into two phases. After separation, the glass vial was capped and stirred. If the carrier candidate had a sufficient encapsulation ability, the organic solvents became emulsions. Deionized (DI) water, mineral oil (M8410, CAS 8042-47-5; Sigma-Aldrich, St. Louis, MO, USA), diethyl ether (Suzuki Chemical Industry), fluorinated oil (Novec^TM^ 7300; 3M Japan Limited, Tokyo, Japan), and perfluorocarbon (PFC)-based solvent (CT-SOLV180; AGC Inc., Tokyo, Japan) were used as the continuous phases for the toluene, chloroform, and dichloromethane droplets, methanol droplets, DMSO droplets, acetonitrile droplets, and acetone droplets, respectively. Saturated saline solution was used as the continuous phase for the THF droplets because these two liquids are immiscible due to salting out. Three nonionic surfactants possessing different HLB values, which indicate the balance in size between the hydrophilic head and hydrophobic tail, were used for stable droplet generation. Surfactant molecules adsorbed to the interface of the dispersed and continuous phases to stabilize it and prevent wetting of the channel by the dispersed-phase solution. A surfactant with a low HLB value is helpful for generating aqueous-in-organic droplets. In contrast, a surfactant with a high HLB value is helpful for generating organic-in-aqueous droplets (Figure 6). Tween 20 (HLB: 16.7, P1379, CAS 9005-64-5; Sigma-Aldrich) was mixed into the DI water. Surflon S-656 (HLB: 8–9; AGC Seimi Chemical Co., Ltd., Chigasaki, Japan) and Span 80 (HLB: 4.3, 37408-32, CAS 1338-43-8; Kanto Chemical Co., Inc., Tokyo, Japan) was mixed into the mineral oil and diethyl ether. Polyvinyl alcohol (PVA, approximate molecular weight 86000, CAS 9002-89-5, Acros Organics; Thermo Fisher Scientific, Waltham, MA, USA) was also used as a surfactant (HLB: 18.0) and channel modifier. The PVA (1, 3 wt %) was added to DI water and the resulting solution was stirred at room temperature for 1 h. The temperature was then gradually increased to 90 °C and the solution was stirred for an additional 1 h. Finally, the temperature was gradually decreased to 65 °C and the solution was left to stir overnight. After dissolution, DI water was added to compensate for any water loss due to evaporation. To prepare saturated saline solution, sodium chloride (Suzuki Chemical Industry) was dissolved in DI water.

### 3.2. Device Design and Fabrication

**Figure 7:**
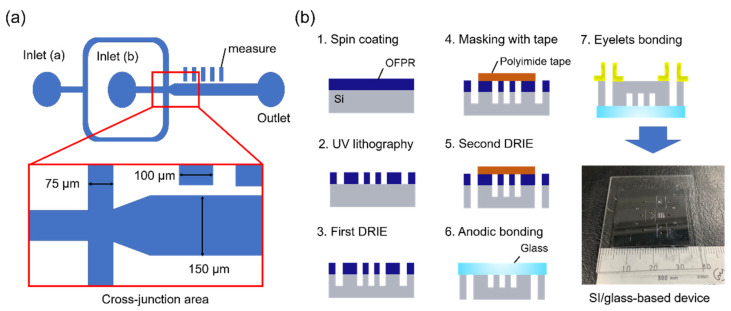
(**a**) Schematic diagram of channel design for the droplet generation device. The widths of the main channel and inlet channel were 150 and 75 μm, respectively, and the channel depth was 100 μm. (**b**) Fabrication process for the droplet generation device with a photograph of the silicon/glass-based device. The major/minor length of the device was about 4/3 cm.

The channel design is depicted in Figure 7a. The dispersed-phase solutions and continuous-phase solutions were injected via inlets (a) and (b), respectively. The channel possessed a tapered shape at the cross-junction area to facilitate droplet formation. The rectangles adjacent to the main channel were designed to allow measurement of the droplet size.

The device fabrication process is presented in Figure 7b. The devices were fabricated via lithography and dry-etching techniques. First, a silicon wafer with a thickness of 525 µm and soda–lime glass with a thickness of 500 µm were cut to obtain square substrates (30 × 30 mm^2^). Positive photoresist (OFPR-800LB, 200cp; Tokyo Ohka Kogyo, Tokyo, Japan) was spin-coated onto the silicon substrate and the channel design was patterned onto the resist via ultraviolet lithography (MA/BA6; SUSS MicroTec, Garching, Germany). The silicon substrate with the patterned resist was dry-etched via deep reactive ion etching (RIE-400iPB; Samco, Kyoto, Japan) to fabricate the channels. After the first etching step, the channel was partially covered with polyimide tape to restrict the etching area prior to a second cycle of etching to form the through holes. After completion of the etching processes, the resist was removed and the glass substrate was bonded to the silicon substrate by anodic bonding to seal the channels. Finally, eyelets were glued onto the substrate to provide the inlets and outlet.

### 3.3. Channel Surface Treatment

The channel surface was modified to alter its wettability to hydrophilic or hydrophobic depending on the type of continuous-phase solution [37,38]. For the microfluidic experiments involving toluene, chloroform, THF, and dichloromethane, a hydrophilic surface was required. This was accomplished by first passing PVA solution through the device channel for 10 min using a glass syringe. The device was then heated at 80 °C for 3 min and the PVA solution remaining in the channel was flushed out using DI water. The device was then heated at 80 °C for 1 h to remove any residual moisture. A hydrophobic surface was needed for the microfluidic experiments involving methanol, DMSO, acetone, and acetonitrile. This surface was achieved by first passing a methanolic solution of trichloro(1*H*,1*H*,2*H*,2*H*-perfluorooctyl)silane (448931-10G, CAS 78560-45-9; Sigma-Aldrich) through the device channel for 10 min using a glass syringe. Then, methanol was then passed through the device for 10 min and the residual methanol was removed by blowing air through the channel followed by heating at 80 °C for 1 h.

### 3.4. Microfluidic Experiment Setup

The device inlets were connected to syringes (1725CX or 1750CX; Hamilton, Reno, NV, USA) via PTFE tubing (500 μm, i.d.) or PVC tubing (1000 μm, i.d.). The dispersed-phase and continuous-phase solutions were injected into the device using syringes and syringe pumps (KD Scientific Inc., Holliston, MA, USA). To reduce instability, the flow rate of the syringe pumps was set to 0.1 µL/min or more and the soft tubes were cut as short as possible. Flow in the device channel was observed by optical microscopy (IX71; Olympus, Tokyo, Japan) and images and movies of the fluids were captured at 2000 fps using a high-speed camera (Fastcam Mini AX; Photron, Tokyo, Japan). When the flow rates of the syringe pumps were changed, videos and photos were captured after waiting a few minutes for the flow rates to stabilize.

## 4. Conclusions

In this work, we have demonstrated droplet generation for wide varieties of organic solvents. Droplets of five common organic solvents (toluene, chloroform, methanol, THF, and DMSO) and three organic solvents highly harmful to resins (acetone, acetonitrile, and dichloromethane) were successfully generated by using appropriate solutions as the continuous phase. The fabricated silicon/glass-based microfluidic device was found to be superior to devices based on resins such as PDMS and thiol–ene-based resin owing to the high resistance of the constituent materials to many organic solvents. The developed device is also expected to be applicable to the generation of droplets of organic solvents with high dissolving power toward resins, such as chlorinated solvents, without any swelling of the device materials. The device also realizes a minimum droplet length of 50 µm and a maximum droplet length of 300 µm. Furthermore, the size of the organic solvent droplets could be controlled by adjusting the flow rates of the two phases, and uniform droplets were obtained for high total flow rates. This latter property is expected to be advantageous for evaluating scaling effects in organic reactions involving microdroplets. The throughput of droplet generation could be increased with optimization of the flow rates, and the device realizes high throughput of droplet generation (2833.3 Hz) at high total flow rate. It was suggested that the addition of a surfactant to the continuous phase and channel surface treatment were helpful for stable organic solvent droplet generation and stabilizing the generated droplets. Comprehensive characterization of the fabricated devices will require further investigations, including contact angle and viscosity measurements. Optimization of surface treatment will be considered as needed. It is necessary to investigate the effect of surfactants on chemical reactions and to optimize the surfactant concentration. The proposed method is anticipated to be applicable to a variety of organic reactions involving microdroplets. Therefore, the results of this study should prove valuable for research into chemical reactions in microdroplets and contribute to the development of novel chemical synthesis strategies.

## Figures and Tables

**Table 1:** Typical flow conditions for generating droplets of five typical organic solvents and their carrier combinations. The sample size of the mean droplet length measurement was 30 droplets.

Dispersed Phase	Continuous Phase	Example Flow Rate at Which Droplets Were Generated			
	Surfactant	Dispersed Phase (µL/min)	Continuous Phase (µL/min)	Mean Droplet Length (µm)	Droplet Generation Rate (Hz)
Toluene	DI water	1	3	286.3 ± 12.2	13.8
	Tween 20 (4 wt %)				
Chloroform	DI water	1	3	183.4 ± 2.7	9.55
	PVA (3 wt %)				
Methanol	Mineral oil	0.3	3	83.9 ± 1.0	23.9
	Surflon S-656 (1 wt %)				
DMSO	Diethyl ether	0.4	8	56.8 ± 1.5	14.7
	Span 80 (2 wt %)				
THF	Saturated saline	0.5	5	102.1 ± 0.8	17.6
	Tween 20 (4 wt %)				

**Table 2:** Typical flow conditions for generating droplets of some organic solvents that are harmful to resins having high chemical resistance and their carrier combinations. The sample size of mean droplet length measurement was 30 droplets.

Dispersed Phase	Continuous Phase	Example Flow Rate at Which Droplets Were Generated			
	Surfactant	Dispersed Phase (µL/min)	Continuous Phase (µL/min)	Mean Droplet Length (µm)	Droplet Generation Rate (Hz)
Dichloromethane	DI water	1	10	109.4 ± 1.75	47.6
	PVA (1 wt %)				
Acetone	PFC-based solvents	1	3	112.3 ± 2.26	27.3
	None				
Acetonitrile	Fluorinated oil	1	3	103.8 ± 3.3	29.6
	None				

**Table 3:** Combinations of continuous-phase and dispersed-phase solutions used in this work.

Dispersed Phase (Droplets)		Continuous Phase (Carrier)		
Liquid	Type (Dipole Moment)	Liquid	Type	Solubility in the Dispersed-Phase Solution
Toluene	Nonpolar sol. (0.36 D)	DI water	Protic polar sol.	0.045 g/100 mL (20 °C)
Chloroform	Nonpolar sol. (1.04 D)	DI water	Protic polar sol.	0.8 g/100 mL (20 °C)
Methanol	Protic polar sol. (1.70 D)	Mineral oil	Nonpolar sol.	Immiscible (No date)
DMSO	Aprotic polar sol. (3.96 D)	Diethyl ether	Nonpolar sol.	Immiscible (No date)
THF	Aprotic polar sol. (1.75 D)	Saturated saline	Protic polar sol.	5.49 g/100 mL (25 °C)
Acetone	Aprotic polar sol. (2.88 D)	PFC-based solvent	Nonpolar sol.	Immiscible (No date)
Acetonitrile	Aprotic polar sol. (3.92 D)	HFE-based oil	Nonpolar sol.	Immiscible (No date)
Dichloromethane	Aprotic polar sol. (1.60 D)	DI water	Protic polar sol.	1.3 g/100 mL (20 °C)

## References

[1] Squires T.M., Quake S.R. (2005). Microfluidics: Fluid physics at the nanoliter scale. Rev. Mod. Phys..

[2] Atencia J., Beebe D.J. (2005). Controlled microfluidic interfaces. Nat. Cell Biol..

[3] Darhuber A.A., Troian S.M. (2005). Principles of microfluidic actuation by modulation of surface stresses. Annu. Rev. Fluid Mech..

[4] Yoon D.H., Tanaka D., Sekiguchi T., Shoji S. (2016). Microfluidic Stamping on Sheath Flow. Small.

[5] McMullen J.P., Jensen K.F. (2010). An Automated Microfluidic System for Online Optimization in Chemical Synthesis. Org. Process. Res. Dev..

[6] Demello A.J. (2006). Control and detection of chemical reactions in microfluidic systems. Nat. Cell Biol..

[7] Craighead H. (2009). Future lab-on-a-chip technologies for interrogating individual molecules. Nanosci. Technol..

[8] El-Ali J., Sorger P.K., Jensen K.F. (2006). Cells on chips. Nature.

[9] Yager P., Edwards T., Fu E., Helton K., Nelson K., Tam M.R., Weigl B.H. (2006). Microfluidic diagnostic technologies for global public health. Nat. Cell Biol..

[10] Psaltis D., Quake S.R., Yang C. (2006). Developing optofluidic technology through the fusion of microfluidics and optics. Nat. Cell Biol..

[11] Christopher G.F., Anna S.L. (2007). Microfluidic methods for generating continuous droplet streams. J. Phys. D Appl. Phys..

[12] Zhu P., Wang L. (2017). Passive and active droplet generation with microfluidics: A review. Lab Chip.

[13] Haneoka M., Shirasaki Y., Sugino H., Aoki T., Arakawa T., Ozaki K., Yoon D.H., Ishii N., Iizuka R., Shoji S. (2011). Microfluidic active sorting of DNA molecules labeled with single quantum dots using flow switching by a hydrogel sol-gel transition. Sens. Actuators B Chem..

[14] Jamshaid A., Igaki M., Yoon D.H., Sekiguchi T., Shoji S. (2013). Controllable Active Micro Droplets Merging Device Using Horizontal Pneumatic Micro Valves. Micromachines.

[15] Link D.R., Anna S.L., Weitz D.A., Stone H.A. (2004). Geometrically Mediated Breakup of Drops in Microfluidic Devices. Phys. Rev. Lett..

[16] Liau A., Karnik R., Majumdar A., Cate J.H.D. (2005). Mixing Crowded Biological Solutions in Milliseconds. Anal. Chem..

[17] Huebner A., Bratton D., Whyte G., Yang M., Demello A.J., Abell C., Hollfelder F. (2009). Static microdroplet arrays: A microfluidic device for droplet trapping, incubation and release for enzymatic and cell-based assays. Lab Chip.

[18] Schneider T., Kreutz J., Chiu D.T. (2013). The Potential Impact of Droplet Microfluidics in Biology. Anal. Chem..

[19] Mashaghi S., Abbaspourrad A., Weitz D.A., Van Oijen A.M. (2016). Droplet microfluidics: A tool for biology, chemistry and nanotechnology. TrAC Trends Anal. Chem..

[20] Shembekar N., Chaipan C., Utharala R., Merten C.A. (2016). Droplet-based microfluidics in drug discovery, transcriptomics and high-throughput molecular genetics. Lab Chip.

[21] Han Z., Li W., Huang Y., Zheng B. (2009). Measuring Rapid Enzymatic Kinetics by Electrochemical Method in Droplet-Based Microfluidic Devices with Pneumatic Valves. Anal. Chem..

[22] Su B., Wang S., Song Y., Jiang L. (2010). A miniature droplet reactor built on nanoparticle-derived superhydrophobic pedestals. Nano Res..

[23] Song H., Chen D.L., Ismagilov R.F. (2006). Reactions in Droplets in Microfluidic Channels. Angew. Chem. Int. Ed..

[24] Theberge A.B., Courtois F., Schaerli Y., Fischlechner M., Abell C., Hollfelder F., Huck W.T.S. (2010). Microdroplets in Microfluidics: An Evolving Platform for Discoveries in Chemistry and Biology. Angew. Chem. Int. Ed..

[25] Fukuda T., Funaki N., Kurabayashi T., Suzuki M., Yoon D.H., Nakahara A., Sekiguchi T., Shoji S. (2014). Real-time monitoring of chemical reaction in microdroplet using fluorescence spectroscopy. Sens. Actuators B Chem..

[26] Macosko E.Z., Basu A., Satija R., Nemesh J., Shekhar K., Goldman M., Tirosh I., Bialas A.R., Kamitaki N., Martersteck E.M. (2015). Highly Parallel Genome-wide Expression Profiling of Individual Cells Using Nanoliter Droplets. Cell.

[27] Koiri R.K., Trigun S.K., Dubey S.K., Singh S., Mishra L. (2007). Metal Cu(II) and Zn(II) bipyridyls as inhibitors of lactate dehydrogenase. BioMetals.

[28] Zhang W., Yang S., Lin Q., Cheng H., Liu J. (2018). Microdroplets as Microreactors for Fast Synthesis of Ketoximes and Amides. J. Org. Chem..

[29] McDonald J.C., Duffy D.C., Anderson J.R., Chiu D.T., Wu H., Schueller O.J.A., Whitesides G.M. (2000). Fabrication of microfluidic systems in poly(dimethylsiloxane). Electrophoresis.

[30] Cygan Z.T., Cabral J.T., Beers K.L., Amis E.J. (2005). Microfluidic Platform for the Generation of Organic-Phase Microreactors. Langmuir.

[31] Geczy R., Sticker D., Bovet N., Häfeli U.O., Kutter J.P. (2019). Chloroform compatible, thiol–ene based replica molded micro chemical devices as an alternative to glass microfluidic chips. Lab Chip.

[32] Wang C., Wang C., Li Z. (2020). Thiol–ene-acrylate Ternary Photosensitive Resins for DLP 3D Printing. J. Photopolym. Sci. Technol..

[33] Anthamatten M., O’Neill S.W., Liu D., Wheler T.M., Vallery R.S., Gidley D.W. (2018). Tunability of Free Volume and Viscoelastic Damping of Thiol–Ene Networks Deep in the Glassy State. Macromolecules.

[34] Piccin E., Ferraro D., Sartori P., Chiarello E., Pierno M., Mistura G. (2014). Generation of water-in-oil and oil-in-water microdroplets in polyester-toner microfluidic devices. Sens. Actuators B Chem..

[35] Lashkaripour A., Rodriguez C., Ortiz L., Densmore D. (2019). Performance tuning of microfluidicflow-focusing droplet generators. Lab Chip.

[36] Bąk A., Podgórska W. (2016). Interfacial and surface tensions of toluene/water and air/water systems with nonionic surfactants Tween 20 and Tween 80. Colloids Surf. A Physicochem. Eng. Asp..

[37] Trantidou T., Elani Y., Parsons E., Ces O. (2017). Hydrophilic surface modification of PDMS for droplet microfluidics using a simple, quick, and robust method via PVA deposition. Microsyst. Nanoeng..

[38] Asano H., Shiraishi Y. (2015). Development of paper-based microfluidic analytical device for iron assay using photomask printed with 3D printer for fabrication of hydrophilic and hydrophobic zones on paper by photolithography. Anal. Chim. Acta.

